# External validation of the Pre-operative Predictive (PROP) score for feasibility of complete cytoreduction in colorectal peritoneal metastases: a single-centre experience

**DOI:** 10.1007/s00423-026-04306-w

**Published:** 2026-10-08

**Authors:** Christoph Steidle, Can Yurttas, Marco Huth, Arnold Radtke, Jan Keck, Philipp Horvath

**Affiliations:** 1 Department of General, Visceral and Transplant Surgery, University Hospital Tübingen, Tübingen, Germany; 2 Department of General, Visceral and Pediatric Surgery, University Medical Center Göttingen, Göttingen, Germany; 3 Department of General, Visceral and Thoracic Surgery, County Hospital Reutlingen, Reutlingen, Germany

**Keywords:** Peritoneal metastases, Colorectal cancer, Cytoreductive surgery, HIPEC, PROP score, Predictive model

## Abstract

**Purpose:**

Colorectal peritoneal metastases (PM) remain a major therapeutic challenge. Cytoreductive surgery (CRS) combined with hyperthermic intraperitoneal chemotherapy (HIPEC) offers prolonged survival in selected patients. Accurate preoperative assessment of resectability is crucial to avoid non-beneficial surgery. The Preoperative Predictive Score (PROP) integrates nine preoperative clinical and radiologic parameters to predict the completeness of cytoreduction (CC). This study aimed to externally validate the PROP Score in an independent European single-centre cohort.

**Methods:**

63 Patients undergoing CRS ± HIPEC for colorectal PM between 2012 and 2022 were retrospectively analysed. PROP scores were calculated from preoperative data and compared with intraoperative CC scores (CC-0, CC-1, CC-2/3). Statistical analysis included χ², Spearman correlation, and receiver operating characteristic (ROC) curve evaluation.

**Results:**

Sixty-three patients were included (CC-0 = 41; CC-1 = 13; CC-2/3 = 9). Median PROP values increased with lower resectability (3.4 vs. 7.2 vs. 10.8; *p* < 0.001). A cutoff ≥ 6 predicted incomplete cytoreduction with 78% sensitivity and 90% specificity (AUC = 0.919). PROP correlated strongly with the Peritoneal Cancer Index (ρ = 0.673; *p* < 0.001). No 30- or 90-day mortality was observed.

**Conclusion:**

The PROP Score reliably predicts the likelihood of complete cytoreduction in colorectal PM using solely preoperative parameters. Moreover it is the first score which is able to preoperatively discriminate patients with a future CC-0 und CC-1 resection. Its external validation supports evidence-based patient selection for CRS ± HIPEC, optimizing surgical decision-making and resource utilization.

## Introduction

Peritoneal metastases (PM) from colorectal cancer (CRC) represent a challenging manifestation of advanced disease with distinct biological and clinical behaviour. Approximately one in five CRC patients present with distant metastases at diagnosis, and in 4–5% of cases the peritoneum is involved, a metastatic pattern historically associated with the poorest outcomes [1–3]. Before the introduction of cytoreductive strategies, treatment relied on palliative systemic chemotherapy, with median survival rarely exceeding twelve months [4].

Over recent decades, cytoreductive surgery (CRS) combined with hyperthermic intraperitoneal chemotherapy (HIPEC) has markedly improved survival in carefully selected patients. Achieving complete macroscopic cytoreduction (CC-0) remains the strongest prognostic factor, underscoring the need for accurate preoperative assessment of resectability [5, 6].

However, despite advances in modern imaging—including contrast-enhanced CT, diffusion-weighted MRI, and PET/CT—small peritoneal implants and subtle serosal disease often remain undetected [7–9]. Consequently, surgeons still frequently encounter unexpectedly high tumor burden at surgical exploration, resulting in incomplete cytoreduction or non-therapeutic laparotomies [10].

Several prognostic and predictive models have been proposed to refine patient selection for CRS and HIPEC [11, 12]. Although clinical criteria such as limited peritoneal disease, absence of signet-ring cell histology, and lack of small-bowel involvement are well established, most available scoring systems rely on intraoperative variables and therefore cannot support true preoperative decision-making.

To address this gap, the Preoperative Predictive (PROP) Score was developed as a structured radioclinical tool integrating nine fully preoperative variables to estimate the likelihood of achieving complete cytoreduction [13]. Building on more than a decade of CRS/HIPEC experience for colorectal PM, the present study aimed to externally validate the PROP Score in an independent European cohort and to evaluate its accuracy in predicting resectability and completeness of cytoreduction.

## Methods

This retrospective, single-centre study included all patients who underwent cytoreductive surgery (CRS) with or without hyperthermic intraperitoneal chemotherapy (HIPEC) for colorectal PM at the University Hospital Tübingen between 2012 and 2022. Data were extracted from a prospectively maintained institutional HIPEC registry. The study was approved by the local ethics committee (reference 720/2017BO2).

### Patient selection

Eligible patients had histologically confirmed CRC adenocarcinoma, radiologic or intraoperative evidence of PM, and complete preoperative datasets including laboratory and imaging findings. Patients with appendiceal primaries, sarcomatosis, or incomplete clinical data were excluded.

### Preoperative imaging

Contrast-enhanced computed tomography (CT) of the thorax and abdomen represented the standard imaging modality for preoperative staging. MRI and PET/CT were not routinely performed for assessment of peritoneal disease. Preoperative CT imaging underwent dedicated radiological review with particular attention to the extent and distribution of peritoneal metastases and imaging-derived variables incorporated into the PROP score, including ascites, omental cake, and small-bowel involvement.

### Surgical procedure

All procedures were performed via midline laparotomy by the same experienced peritoneal malignancy team following Sugarbaker’s peritonectomy principles [14]. The intraoperative tumor burden was quantified using the Peritoneal Cancer Index (PCI) [15]. Completeness of cytoreduction (CC) was classified as CC-0 (no visible disease), CC-1 (residual nodules < 2.5 mm), CC-2 (2.5 mm–2.5 cm), or CC-3 (> 2.5 cm).

HIPEC was performed using a closed-abdomen technique at 42 °C with either oxaliplatin (460 mg/m²) or cisplatin (100 mg/m²), according to institutional treatment protocols that evolved during the study period.

### PROP score calculation

The Preoperative Predictive (PROP) Score was calculated as described by Yong et al. [13]. It integrates nine preoperative clinical and radiologic variables: previous suboptimal resection, ≥ 4 cycles of systemic chemotherapy, poor differentiation (G3), elevated tumor markers (CEA > 5 µg/L or CA 19 − 9 > 37 U/mL), meteoristic abdomen, palpable mass, ascites on imaging, small-bowel involvement, and omental cake (Table 1).

**Table 1:** PROP score variables

*N*.	Variable	Points
1	Previous suboptimal resection	1
2	≥ 4 cycles of systemic chemotherapy	1
3	Poor differentiation (G3)	1
4	Elevated tumor markers	2
5	Meteoristic abdomen	2
6	Palpable mass	2
7	Ascites on imaging	2
8	Small-bowel involvement	2
9	Omental cake	2

[13]

### Statistical analysis

All analyses were conducted using SPSS v27 (IBM Corp. Version 27, 2020)). Continuous variables are reported as mean ± standard deviation (SD) or median (range), and categorical variables as counts and percentages. Between-group comparisons employed the χ² test, Fisher’s exact test, or the Kruskal–Wallis test, as appropriate. Spearman’s rank correlation coefficient (ρ) was used to assess the relationship between PROP and PCI. Diagnostic performance was evaluated by receiver operating characteristic (ROC) curve analysis. Statistical significance was defined as *p* ≤ 0.05.

## Results

### Cohort characteristics

Sixty-three patients undergoing CRS with or without HIPEC for CRC PM were included. Completeness of cytoreduction was CC-0 in 41 (65.1%), CC-1 in 13 (20.6%), and CC-2/3 in 9 patients (14.3%). Median age was 56 years (22–78) and comparable across groups. CC-0 and CC-1 patients showed a slight female predominance, whereas CC-2/3 patients were mainly male. All patients had favorable baseline functional status (ECOG 0–1; ASA II–III), and no patient presented with ECOG ≥ 2 or ASA IV (Table 2).

**Table 2:** Cohort characteristics (significant *p* < 0.05)

Patient variable	CC-0	CC-1	CC-2/3	*p*-value
Number (n)	41	13	9	0.21
Age (years), median (range)	56 (22–78)	56 (29–71)	56 (29–77)	0.99
Sex (m: f)	18:23	4:9	7:2	0.21
ECOG (0/1/2)	24/17/0	8/5/0	6/3/0	0.99
ASA (1/2/3)	0/24/17	1/8/4	1/6/2	0.64
Primary tumor location				0.09
Right/Left/Transverse	9/28/4	8/5/0	3/5/1	
Histology				**0.019**
Adeno/mucinous/signet ring	34/7/1	7/2/4	4/3/2	
Grading G1/G2/G3				**0.004**
	0/28/13	0/2/11	0/2/7	
PCI-score (median, range)	9.85 (2–23)	17.6 (5–28)	29.6 (18–39)	**< 0.001**
Disease setting				0.56
Synchronous/metachronous	22/19	6/7	6/3	
Chemotherapy before HIPEC				0.37
(0/4–8/>8)	5/20/16	2/6/5	0/4/5	
HIPEC				**< 0.001**
OX-5FU/Cisplatin	22/19	11/2	0/0	
HIPEC duration (min)				**< 0.001**
30/60	22/19	11/2	0/0	
Operation time (median, range)	366 (151–688)	389 (189–651)	184 (33–331)	**< 0.001**
RAS status				0.18
WT/mutated	17/24	9/4	3/6	
BRAF status				0.83
WT/mutated	39/2	11/2	8/1	
PROP score (median, range)	3.41 (1–8)	7.23 (3–13)	10.77 (7–15)	**< 0.001**
PROP score ≥ 6 (n)	4	9	9	**< 0.001**
PROP score < 6 (n)	37	4	0	**< 0.001**
Clavien-Dindo >3a (n)	4	4	0	0.17
Re-operations n (%)	3/41 (7.3%)	4/13 (30.7)	0 (0)	**0.04**

### Oncologic and molecular characteristics

Left-sided primaries were more common in CC-0 (68%), while right-sided tumors predominated in CC-1 (62%). Histologic subtypes associated with poorer prognosis—mucinous and signet-ring were more frequent among patients with incomplete cytoreduction. Poor differentiation strongly correlated with limited resectability: G3 tumors were present in 32% of CC-0, 85% of CC-1, and 78% of CC-2/3 patients. Most patients received preoperative chemotherapy; only 12% (CC-0) and 15% (CC-1) were chemotherapy-naïve, while all CC-2/3 patients had prior systemic treatment [Table 1]. RAS mutations, which were rare across all groups, occurred in 58% of CC-0, 31% of CC-1, and 67% of CC-2/3 patients.

### Disease distribution and tumor burden

Tumor burden differed markedly between groups. Median PCI increased stepwise with lower resectability (*p* < 0.001): 10 for CC-0, 18 for CC-1, and 30 for CC-2/3 patients. Six CC-0 patients presented with PCI > 17, whereas all CC-2/3 patients had PCI ≥ 18. Disease presentation was synchronous in 22 CC-0, 6 CC-1, and 6 CC-2/3 cases.

### Postoperative outcomes

Major postoperative complications (Clavien–Dindo ≥ 3a) occurred in 8 CC-0 (19.5%) and 7 CC-1 (53.8%) patients, but in none of the CC-2/3 patients who did not undergo complete cytoreduction. Reoperation was required in 3 CC-0 (7.3%) and 4 CC-1 (30.7%) patients, due to fascial dehiscence or anastomotic leak. No 30- or 90-day mortality occurred in the entire cohort.

### PROP score performance

**Fig. 1:**
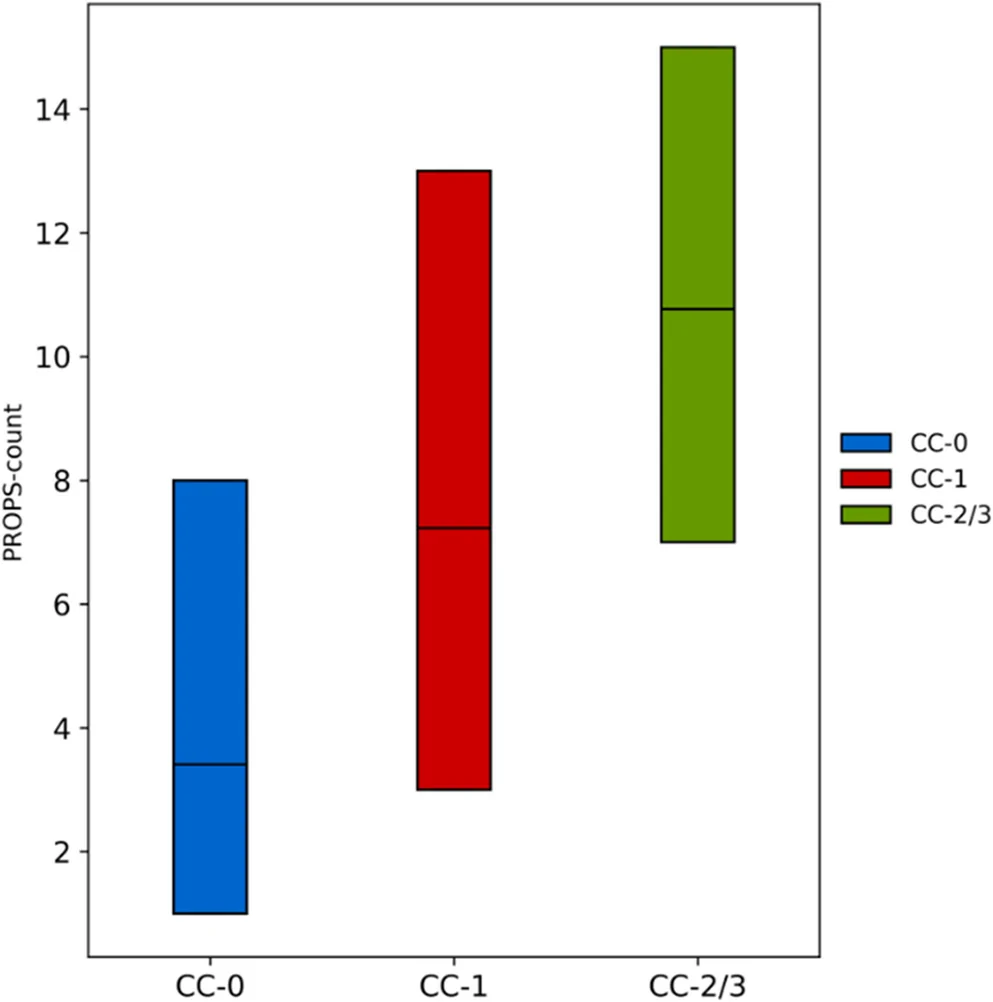
Boxplot showing the stepwise increase of the PROP-score with declining resectability

PROP scores showed a strong stepwise increase with decreasing resectability (*p* < 0.001). Median values were 3 (range, 1–8) for CC-0, 7 (range, 3–13) for CC-1, and 11 (range, 7–15) for CC-2/3 (Fig. 1). A score ≥ 6 identified 9.8% of CC-0, 69.2% of CC-1, and 100% of CC-2/3 patients. The discriminatory capacity of the score was further confirmed by ROC analysis. This yielded an area under the curve of 0.919, indicating excellent diagnostic performance. The 95% confidence interval (0.849–0.988) underscores the robustness of these findings. The ROC curve demonstrated clear separation between patients undergoing CC-0 resection and the other groups. Ascites, omental cake, and small-bowel involvement were the parameters most strongly associated with incomplete cytoreduction and showed a high statistical significance between CC-0 and CC-2/3 but not between CC-1 and CC-2/3. As only parameter, the presence of an omental cake, was statistically significant different between CC-0 and CC-1 (Table 3).

**Table 3:** PROP variables most strongly associated with incomplete cytoreduction (significant *p* < 0.05)

Variable	*p*-value
Ascites	
CC-0 vs. CC-1	0.42
CC-0 vs. CC-2/3	**0.003**
CC-1 vs. CC-2/3	0.06
Omental cake	
CC-0 vs. CC-1	**0.00003**
CC-0 vs. CC-2/3	**< 0.00000**
CC-1 vs. CC-2/3	0.19
Small bowel involvement	
CC-0 vs. CC-1	0.39
CC-0 vs. CC-2/3	**0.0005**
CC-1 vs. CC-2/3	0.06

## Discussion

This study provides the first independent validation of the Preoperative Predictive (PROP) Score outside the original cohort and confirms its strong performance in estimating cytoreductive feasibility in colorectal peritoneal metastases (CRC-PM). PROP values increased consistently across surgical outcome groups (CC-0 to CC-2/3), and a threshold of ≥ 6 demonstrated excellent discriminatory capacity (AUC 0.919). Despite the retrospective, single-centre design, the reproducibility of these findings compared with the index study supports the robustness and potential generalisability of the PROP score.

A key contribution of the present study is the confirmation that the PROP score represents, to our knowledge, the first clinically applicable scoring system capable of predicting cytoreductive outcomes using exclusively preoperative variables, without reliance on intraoperative findings. This distinguishes PROP fundamentally from existing clinical scores and aligns it conceptually with emerging predictive approaches such as artificial intelligence (AI)-based models [12, 16, 17].

Achieving complete cytoreduction (CC-0) remains the strongest determinant of survival in peritoneal surface malignancies (PSM), including CRC-PM [18–20]. Importantly, contemporary evidence has clarified that even minimal residual disease (CC-1) is associated with significantly inferior survival compared with CC-0, challenging the historical perception of CC-1 as “near-complete” cytoreduction [21]. Consequently, the ability to accurately distinguish patients who will achieve CC-0 from those likely to have CC-1 or CC-2/3 resections is of central clinical importance.

However, accurate preoperative prediction of cytoreductive outcomes remains a major unmet need. Conventional imaging techniques, particularly CT-based assessment, demonstrate limited sensitivity for peritoneal disease and frequently underestimate tumour burden, especially in the small bowel and mesentery [22]. Even staging laparoscopy, often considered the diagnostic gold standard, may underestimate disease extent in up to 25–30% of cases [23]. These limitations highlight the need for robust, reproducible, and truly preoperative prediction tools.

In this context, the recently published scoping review on AI-based prediction of cytoreduction completeness provides important insight into the evolving landscape of predictive modelling in PSM. Across nine included studies, AI models—particularly those integrating radiomics with clinical variables—achieved AUC values ranging from 0.70 to 0.98, demonstrating promising predictive performance. Notably, multimodal models combining imaging-derived radiomic features with clinical data consistently outperformed single-modality approaches, and deep learning frameworks such as the DeAF model achieved robust multicentre validation with AUCs around 0.90 [17].

Despite these encouraging results, several critical limitations of AI-based approaches must be considered. First, most models rely on complex radiomic feature extraction or deep learning architectures, requiring advanced computational infrastructure, specialised software, and expert input for image segmentation. Second, external validation remains limited, with only a minority of studies demonstrating true multicentre generalisability. Third, and perhaps most importantly, model explainability is insufficient, with most AI systems functioning as “black boxes” that do not provide transparent reasoning for individual predictions. This lack of interpretability represents a major barrier to clinical adoption.

Against this background, the PROP score occupies a unique and complementary position. While AI-based models aim to maximise predictive accuracy through complex data integration, PROP provides a simple, transparent, and fully explainable scoring system based on nine routinely available preoperative variables. Unlike radiomics or deep learning approaches, PROP does not require specialised imaging processing, segmentation, or computational expertise, making it readily applicable in routine clinical practice across a wide range of institutions.

Importantly, our findings demonstrate that PROP is not only capable of distinguishing resectable from unresectable disease, but also accurately differentiates between CC-0 and CC-1/2/3 outcomes preoperatively. This represents a critical advance, as prior models—including many AI-based approaches—have primarily focused on broader endpoints such as resectable versus unresectable disease or residual tumour thresholds (e.g., < 1 cm), rather than the clinically decisive distinction between CC-0 and CC-1. Indeed, the AI literature highlights that predictive performance often decreases substantially when stricter endpoints such as complete cytoreduction (R0/CC-0) are used, underscoring the significance of the PROP score’s discriminatory capacity in this setting.

From a clinical perspective, this distinction has direct therapeutic implications. Patients predicted to achieve CC-0 are most likely to benefit from CRS and HIPEC, whereas those with a high probability of CC-1 or incomplete cytoreduction may be better managed with systemic therapy or alternative strategies prior to secondary surgery. In our cohort, PROP demonstrated excellent stratification, with low scores strongly associated with CC-0 resection and high scores reliably identifying unresectable disease. Notably, a PROP score ≥ 10 would have correctly identified all patients with CC-2/3 resections, thereby potentially avoiding non-therapeutic laparotomies.

The clinical relevance of avoiding futile surgery cannot be overstated. CRS and HIPEC are associated with substantial morbidity, resource utilisation, and impact on quality of life [24]. AI-based models similarly aim to reduce unnecessary surgery by improving preoperative prediction; however, their current limitations in accessibility and interpretability restrict widespread implementation. In contrast, PROP offers an immediately deployable solution that can be integrated into multidisciplinary decision-making without additional infrastructure.

Rather than competing approaches, PROP and AI-based models should be viewed as complementary strategies. PROP may serve as a first-line, easily applicable triage tool, identifying patients with a high or low likelihood of achieving CC-0. In more complex or borderline cases, advanced AI models incorporating radiomics or deep learning could provide additional refinement. Future research may explore hybrid approaches combining PROP variables with radiomic or AI-derived features to further enhance predictive accuracy while maintaining clinical interpretability.

The PROP score also aligns well with current guideline-based selection criteria. International recommendations emphasise tumour burden, small-bowel involvement, and tumour biology as key determinants of resectability [25, 26]. PROP integrates several of these factors into a structured scoring system, thereby complementing guideline-based decision-making and potentially improving standardisation across centres.

A particular challenge in the preoperative assessment of peritoneal disease remains the accurate evaluation of small-bowel and small-bowel mesenteric involvement. Although contrast-enhanced CT represents the standard imaging modality for preoperative staging, discrepancies between radiological and intraoperative assessment of the PCI have consistently been reported. In a multi-institutional study by Esquivel et al., CT-based PCI underestimated the intraoperative extent of peritoneal disease in approximately one third of patients, with significant inaccuracies in lesion-size assessment particularly observed in the distal jejunum and distal ileum [27]. These limitations are of particular clinical relevance, as diffuse involvement of the small bowel or its mesentery represents one of the principal factors precluding complete cytoreduction. Sugarbaker et al. subsequently emphasized that small-volume serosal implants, mesenteric disease, and multifocal visceral involvement may remain difficult to identify reliably by CT and that radiological criteria predicting incomplete cytoreduction have not yet been sufficiently standardized [28]. Consequently, both under- and, less frequently, overestimation of small-bowel disease may occur, potentially resulting in misclassification of the true intraoperative disease burden. This limitation is particularly relevant to the PROP score, as small-bowel involvement constitutes one of its nine preoperative variables. Thus, PROP should not be interpreted as overcoming the inherent diagnostic limitations of preoperative imaging, but rather as a structured tool integrating the clinical and radiological information available before surgical exploration. Nevertheless, uncertainty regarding small-bowel involvement remains an inherent limitation of the present analysis and should be considered when applying the PROP score in individual patients. Further limitations of this study must be acknowledged. The retrospective design and moderate sample size may introduce selection bias. Furthermore, the study population was limited to colorectal primaries, restricting generalisability to other tumour entities. Prospective, multicentre validation studies are required to confirm the performance of the PROP score across diverse clinical settings and imaging protocols.

In conclusion, this study provides independent external validation of the PROP score and supports its potential value as a fully preoperative tool for estimating cytoreductive outcomes in patients with CRC-PM. The observed discrimination between CC-0 and CC-1/2/3 outcomes suggests that PROP may complement established clinical and radiological criteria in preoperative patient selection. However, given the retrospective single-centre design, the moderate sample size, and the inherent limitations of conventional imaging, particularly in the assessment of small-bowel involvement, these findings should be interpreted with appropriate caution. Rather than serving as a stand-alone decision-making tool, PROP may provide an easily applicable and transparent adjunct to multidisciplinary assessment. Prospective multicentre validation using standardized imaging protocols and independent radiological assessment is required to confirm its predictive performance and define its role in clinical practice.

## Data Availability

Data were retrieved from a prospective maintained HIPEC registry at the operating institution. The dataset is aligned with personal data and cannot be shared publicly.
